# Population network structures, graph theory, algorithms to match subgraphs may lead to better clustering of households and communities in epidemiological studies: a response

**DOI:** 10.1017/S0950268819002243

**Published:** 2020-01-10

**Authors:** A. Trickey, A. Sood, V. Midha, W. Thompson, C. Vellozzi, S. Shadaker, V. Surlikar, S. Kanchi, P. Vickerman, M. T. May, F. Averhoff

**Affiliations:** 1 Population Health Sciences, University of Bristol, Bristol, UK; 2 National Institute of Health Research (NIHR) Health Protection Research Unit (HPRU) in Evaluation of Interventions at the University of Bristol, Bristol, UK; 3 Dayanand Medical College, Civil lines, Tagore Nagar, Ludhiana, Punjab, India; 4 Centers for Disease Control and Prevention, Atlanta, GA, USA; 5 MSD India Pvt. Ltd., Mumbai, India

We would like to thank Professor Rao for his letter describing the graphical structure of clustering through networks [1]. We agree that network structure data are a useful way to understand the patterns of transmission. When combined with mathematical modelling, these methods also allow for measuring the effect of interventions such as by Metzig *et al*. when looking at the transmission of hepatitis C virus (HCV) among networks of people who inject drugs (PWID) [2]. Adding attribute-level information to each vertex, as described in the letter by Professor Rao, is an interesting development to these network structures and could be of use for determining which attributes are associated with prevalent infections in certain circumstances.

Regarding our work examining clustering of prevalent HCV infections in a general population setting in Punjab state, India [3], which used data from a seroprevalence survey [4], we do not believe the method described by Professor Rao would be applicable as we do not have detailed data on the social networks of the study participants. The method outlined would be ideal when investigating bloodborne infection patterns, such as HCV, among PWID. Globally, unsafe injecting practices among PWID are estimated to cause approximately 40% of ongoing HCV infections and are clearly an area in need of immediate attention [5]. In settings with a high prevalence of injection drug use, the transmission is most likely to have occurred through the sharing of unsafe injecting equipment, but in more generalised epidemic settings such as Punjab, this is not the case due to other competing risks. For example, HCV transmission could occur between two people who share a particular medical practitioner using unsterilised equipment and therefore the two individuals may share few, if any, clearly defined social networks. In such circumstances, the use of network models would be highly complicated and would require very granular data that are usually unavailable.

We believe our study adds to the evidence base for factors associated with prevalent HCV infections in Punjab state, India, which can be of use for those setting up screening programmes. Specifically, our study found that clustering of HCV antibody positivity does occur within households. To validate this, we have subsequently investigated the 18 households containing ≥2 HCV-infected people, where at least two members had successful genotype tests. Of these households, 14 had two or more members with the same genotype. Punjab has a diverse genotype distribution, with 61% of those successfully tested in our survey having genotype 3, 28% genotype 1 and 11% genotype 4. The genotype distributions among the individuals from the households with multiple infections were not dissimilar to those of the overall sample (50% genotype 3, 28% genotype 1, 23% genotype 4). Considering the distribution of genotypes in this setting, where none completely dominates, we would expect to see more discordant genotypes in households if clustering was not occurring. This reinforces the recommendation that when an HCV-infected person is identified, testing of other members of the household would be prudent, as the risk of HCV infection is elevated for individuals in those households.

We agree with Professor Rao that both our current work as well as future work provide the potential to organise and collect data to develop more complex epidemiologic models. Due to reports indicating injecting drug use could be an important route of HCV transmission in Punjab [6, 7], we advise those designing future studies among PWID in this setting to try to account for social network structure and apply the methods outlined in the letter by Professor Rao. The collection of high-quality empirical data is key to informing models.
